# The Cryptic African Wolf: *Canis aureus lupaster* Is Not a Golden Jackal and Is Not Endemic to Egypt

**DOI:** 10.1371/journal.pone.0016385

**Published:** 2011-01-26

**Authors:** Eli Knispel Rueness, Maria Gulbrandsen Asmyhr, Claudio Sillero-Zubiri, David W. Macdonald, Afework Bekele, Anagaw Atickem, Nils Chr. Stenseth

**Affiliations:** 1 Centre for Ecological and Evolutionary Synthesis, Department of Biology, University of Oslo, Oslo, Norway; 2 Wildlife Conservation Research Unit, Department of Zoology, University of Oxford, Oxford, United Kingdom; 3 Biology Department, Science Faculty, Addis Ababa University, Addis Ababa, Ethiopia; Natural History Museum of Denmark, Denmark

## Abstract

The Egyptian jackal (*Canis aureus lupaster*) has hitherto been considered a large, rare subspecies of the golden jackal (*C. aureus*). It has maintained its taxonomical status to date, despite studies demonstrating morphological similarities to the grey wolf (*C. lupus*). We have analyzed 2055 bp of mitochondrial DNA from *C. a. lupaster* and investigated the similarity to *C. aureus* and *C. lupus*. Through phylogenetic comparison with all wild wolf-like canids (based on 726 bp of the Cytochrome *b* gene) we conclusively (100% bootstrap support) place the Egyptian jackal within the grey wolf species complex, together with the Holarctic wolf, the Indian wolf and the Himalayan wolf. Like the two latter taxa, *C. a. lupaster* seems to represent an ancient wolf lineage which most likely colonized Africa prior to the northern hemisphere radiation. We thus refer to *C. a. lupaster* as the African wolf. Furthermore, we have detected *C. a. lupaster* individuals at two localities in the Ethiopian highlands, extending the distribution by at least 2,500 km southeast. The only grey wolf species to inhabit the African continent is a cryptic species for which the conservation status urgently needs assessment.

## Introduction

**Figure 1:**
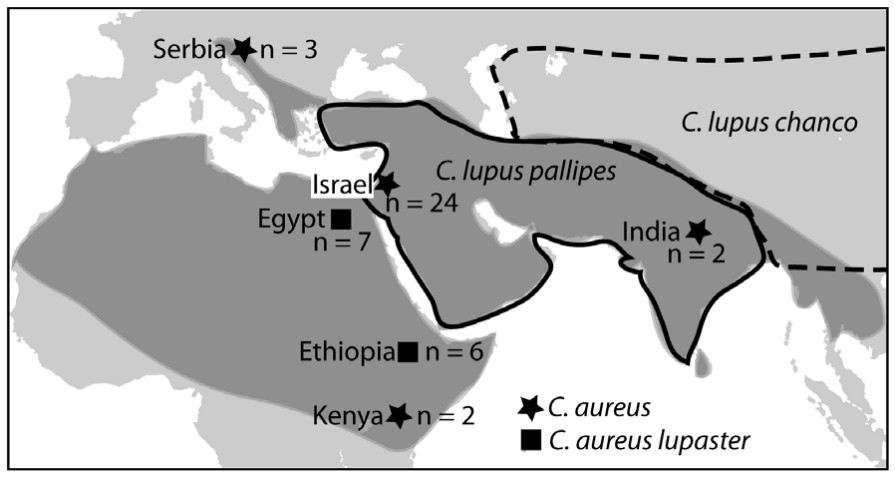
Map of distribution ranges and samples. The distribution area of the golden jackal in Africa and Eurasia is shaded dark grey. The samples analysed are shown as symbols: squares  =  *C.a. lupaster* and stars  =  *C. aureus*. The number next to the symbols equals the number of samples from each site. The approximate border of the distribution areas of *C. lupus pallipes* and *C. lupus chanco* are delineated by black and slashed lines respectively.

The golden jackal (*Canis aureus*; Linneaus 1758) is currently considered a monophyletic species among the wolf-like canids [1], [2]. Found throughout north and east Africa, the Middle East, southeastern Europe, and central, southern and western Asia (Figure 1) [3], [4] this species shows large morphological and ecological intra-species variability [e.g. 5]–[7].

The Egyptian jackal (*Canis aureus lupaster*; Hemprich and Ehrenberg 1833) is, as per conventional taxonomy, considered a subspecies of the golden jackal, although the similarity of the skulls of certain North African jackals to that of the Indian wolf (*Canis lupus pallipes*) had already been noted by Thomas Huxley as early as 1880 [8]. The Egyptian jackal overlaps in size with the grey wolf (*Canis lupus*), being larger and more long-limbed than the Holotype *Canis aureus*, and its cranial features differ from other golden jackals [9]. Nassef [10] investigated the relative relationship between Egyptian and Israeli jackals and found through phylogenetic analysis of a segment of the Cytochrome b gene (Cyt *b*) that the Egyptian jackal was more similar to grey wolves. Their data were, however, very scarce and the conclusion was to retain the Egyptian jackal as a *C. aureus* subspecies. Here we provide more data and challenge this conclusion.

The grey wolf has a Holarctic distribution with as many as 30 subspecies recognized [4], although wolves throughout their enormous range have been shown to be genetically very similar [11]. Recently, molecular analysis showed that two subspecies, the Indian wolf (*C. l. pallipes*) and the Himalayan wolf (*C. l. chanco/laniger*) represent ancient wolf lineages that merit species status [12], [13]. We will refer to these three main wolf lineages as the grey wolf species complex.

The grey wolf currently extends to the Sinai Peninsula, but is not found in mainland Africa; the presumed closest relative in this continent is the rare and endangered Ethiopian wolf (*Canis simensis*) [14]. This species is endemic to the Ethiopian highlands where they are sympatric with golden jackals.

During a field study of the Ethiopian wolf in Central Ethiopia we noticed that some golden jackals differed slightly in their appearance from golden jackals elsewhere, in being larger, more slender and sometimes with a more whitish coloration. A wolf-like animal photographed in Eritrea in 2004 was speculated to be an Egyptian jackal [15]. Thus we decided to investigate these highland golden jackals and sequenced 2055 base pairs (bp) of the mitochondrial DNA (mtDNA) from specimens collected in Ethiopia. Through comparisons with other species of wolf-like canids, we present evidence suggesting that:

- *C. a. lupaster* is present in the highlands of Ethiopia, effectively expanding the taxon's trange by at least 2,500 km to the southeast.
- *C. a. lupaster* is not a golden jackal and should be placed within the grey wolf species complex.
- *C. a. lupaster* most likely represents an ancient wolf lineage that colonized Africa prior to the radiation of the Holarctic wolf and as such should be reclassified as the African wolf.

## Materials and Methods

Figure 1 shows the geographic distribution of the samples analysed.

Samples (faeces) representing at least five individuals (as verified by genotyping) were obtained from the Menz region of central Ethiopia. One of the individuals was filmed during defecation, linking with certainty the larger jackal morphotype with the DNA sequence. An additional tissue sample was obtained from a road kill in Arsi in southeast Ethiopia. DNA samples of golden jackals from Serbia were provided by Zachos [16]. The following primers were used to amplify fragments of the mtDNA: 12S rRNA: gene L01091 and H01478 [17],16S rRNA gene: W16S_F (5′- TCTTGAATAGGATTGCGCTG -3′) and W16S_R (5′- CGGGAATGCCACAATAAGAC-3′), Cyt *b* gene: cytb-1 and cytb-2 [18], D-loop: Thr-L 15926, DL-H 16340 [11]. Sequences of Egyptian and Israeli jackals were obtained from Nassif [10]. Sequenc data for other taxa analysed were downloaded from GenBank. Accession numbers for all sequences analysed are given in Table 1.

**Table 1:** Accession number for sequences analyzed for each DNA fragment.

Taxon	Cyt *b*	D-loop	16S	12S
*C. a. lupaster* (Ethiopia)	HQ845258	HQ845259	HQ845257	HQ845256
*C. lupus* - grey wolf	AM711902		NC_009686	GU174606
*C. lupus* - grey wolf (Canada)		DQ480508		
*C. lupus* - grey wolf (Sweden)		DQ480504		
*C. lupus* - gray wolf (Saudi-Arabia)		DQ480506		
*C. lupus* - gray wolf (Japan)		AB480742		
*C. l. pallipes* – Indian wolf	AY291432	AY289984	AY289968	
*C. l. chanco* - Himalayan wolf	AY291431	AY289995	AY289963	GQ374438
*C. aureus* -golden jackal (India)	AY2911433	AY289997	AY289970	DQ102371
*C. aureus* -golden jackal * (Kenya)	AF028138/62			
*C. aureus* - golden jackal (Serbia)		HQ845260		
*C. latrans* - coyote	DQ480511			
*C. simensis* -Ethiopian wolf	HQ845261	HQ845262		
*C. mesomelas* – black-backed jackal	AF028143/66			
*C. adustus* – side-striped jackal	AF028136/60			
*Cuon alpinus* - dhole	GU063864			
*Lycaon pictus* – African wild dog	AF028147/71			
*Vulpes vulpes* -red fox	AB292742			
*Alopex lagopus* - Arctic fox	AY598511			

The optimal model for the phylogenetic analysis selected for the Cyt *b* region by Modeltest [19] following Akaike's information criterion (AIC) was TVM+G and the shape parameter of the gamma distribution was 0.2002. PAUP* 4.0 [20] was used for computing maximum likelihood (ML) and neighbor joining (NJ) phylogenies using red fox (*Vulpes vulpes*) and Arctic fox (*Alopex lagopus*) as outgroups.

## Results

**Figure 2:**
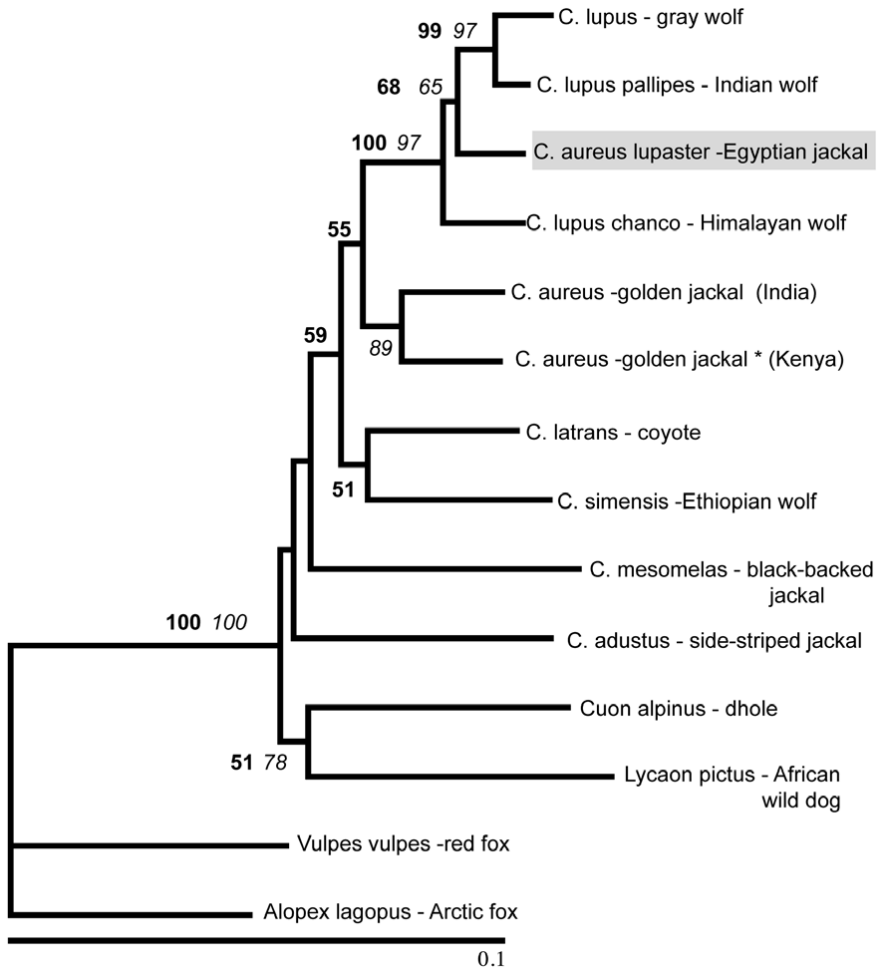
Phylogenetic tree displaying the relationship among all wild wolf-like canids. Phylogeny (Maximum Likelihood) of wolf-like canids based on 726 bp of the Cyt *b* gene. Bootstrap values (>50%) from 10,000 replicates are given next to the branches, **NJ**, *ML*. * This sequence of *C. aureus* which was published by Wayne *et al.* [1] consists of two fragments whereof the first (394 bp bp) show most resemblance to the side-striped jackal while the second (332 bp) differ by 6 point mutations compared to the Eurasian golden jackal.

**Figure 3:**
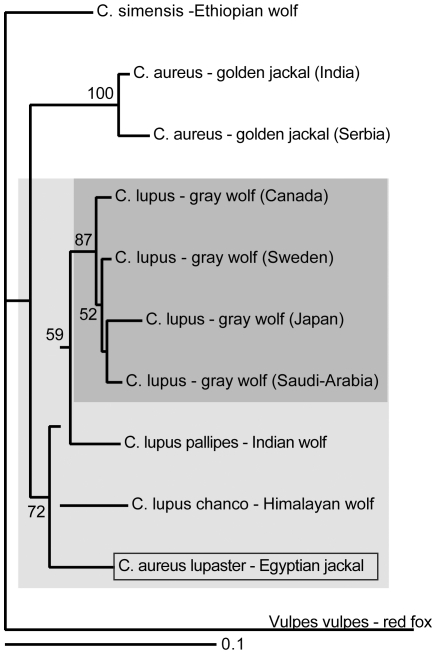
Phylogeny (NJ) based on 317 bp of the mtDNA D-loop region. Bootstrap values (>50%) from 10,000 replicates are given next to the branches. The grey wolf species complex forms a monophyletic group (shaded in light grey), a subgroup consisting of four taxa representing the Holarctic wolf is shaded in dark grey.

A 365 bp fragment of the Cyt *b* gene was comparable between our data and those of Nassef (2003), representing the only Egyptian jackal data available. The Ethiopian and the Egyptian jackals differed by only two transitions. For comparison, the number of segregating sites separating the Ethiopian and Israeli golden jackals was 18. Based on the sequence similarity we defined our Ethiopian golden jackal samples as C. *a. lupaster* for further analysis. The Serbian jackal sequence was identical to the Israeli jackal haplotype, differing from the Indian jackal by four transitions. Figure 1 shows the geographical distribution *C. aureus* and *C. a. lupaster* specimens analysed. Figure 2 displays the phylogenetic relationship among all the wild-living species of wolf-like canids. The tree is based on 726 bp of the Cyt *b* gene for which corresponding sequence data for all of the species exist. The *C. a. lupaster* clusters within the grey wolf species complex, a clade with strong statistical support (100/97% of 10,000 NJ/ML bootstrap replicates). Table 2 presents the number of pairwise differences observed between *C. a. lupaster* as compared to golden jackals and wolves in 1096 bp of the Cyt *b* gene as well as fragments of three other mtDNA regions: the conserved 12S (353 bp) and 16S (289) rRNA genes and the hypervariable D-loop (317 bp). Generally, *C. a. lupaster* shows closer resemblance to wolves than to jackals, corroborating the result presented in Figure 2. Figure 3 shows a NJ phylogeny based on D-loop sequence fragments from *C. a. lupaster*, golden jackals, various grey wolves, Indian and Himalayan wolves, and Ethiopian wolves. The grey wolf species complex, including *C. a. lupaster*, formed a monophyletic clade that was supported by 72% of the 10,000 bootstrap replicates. Within this clade, the sequences of four grey wolves sampled in widespread localities (North America, Northern Europe, Eastern Asia and the Middle East) clustered in a group supported by 87% of the bootstrap replicates. The tree topology suggests that this Holarctic group has a more recent origin than the other genealogical lineages of the grey wolf species complex.

**Table 2:** Number of pairwise differences.

	*C. aureus lupaster*			
	12S 353 bp	16S 289 bp	Cyt *b* 1096 bp	D-loop 317 bp
*C. aureus*	8	6	66	27
*C. lupus*	3	6	37	16
*C. l. pallipes*	NA	6	36	17
*C.l. chanco*	3	5	35	20

## Discussion

Our results show that *C. a. lupaster*, the African wolf, inhabits at least two different localities in Ethiopia, approximately 2,500 km southeast of Egypt. Furthermore, comparisons of DNA sequence data (Table 2, Figure 2) demonstrate that *C. a. lupaster* shows more similarity to *C. lupus* than to *C. aureus*. The grey wolf species complex, including *C. a. lupaster*, forms a monophyletic group with strong statistical support (Figure 2, Figure 3). To consider *C. a. lupaster* as a subspecies of *C. aureus* would imply that the golden jackal is a polyphyletic taxon. It is also evident from Figure 2, as well as from other phylogenetic studies [1], [13], [21], that the golden jackal does not form a monophyletic group with the two other jackal species, the side-striped jackal (*C. adustus*) and the black-backed jackal (*C. mesomelas*). There is thus reason to question whether the colloquial name jackal has any taxonomic integrity.

The Cyt *b* gene is a marker commonly used to distinguish among mammalian species and >5% divergence is typically observed between morphologically recognized species [22]. The Himalayan wolf and the Indian wolf diverge from the Holarctic grey wolf by 1.2% and 2.5% (332 bp) respectively [13]. The divergence between *C. a. lupaster* and *C. lupus* is 4.0% for the equivalent sequence fragment, while it is 2.4% between *C. a. lupaster* and the Himalayan wolf. Wayne [1] estimated a mtDNA molecular clock applicable across Canidae (1.3–1.7% per million years). Estimates of evolutionary time based on restricted data tend to be highly uncertain, but based on sequence divergence we find it is reasonable to consider *C. a. lupaster* as a distinct taxon within the grey wolf species complex.

A high level of polymorphism and homoplasy has been reported for the D-loop region, within and between canid species [13]. The grey wolf species complex was nevertheless statistically supported in our phylogenetic reconstruction (Figure 3), as was the Holarctic sub-clade comprising wolves from North America, Eastern Asia, the Middle East and Northern Europe. The phylogenetic reconstruction suggests that the Indian, Himalayan and the African wolf existed as distinct lineages before the radiation of the Holarctic wolf. Figure 1 shows that there is a geographical ‘continuum’ in the present distribution and one could imagine that a common ancestor of the three species migrated into Africa during Pleistocene (2.6 mill-12,100 years ago).

The golden jackal is listed as a species of Least Concern by the IUCN [7]. In Ethiopia the golden jackals, which presently includes the cryptic African wolves, are systematically persecuted because of their threat to livestock. Furthermore, although the Egyptian jackal is supposedly extremely rare, it is not protected.

All of the Ethiopian samples we analyzed were *C.l. lupaster*, however, a thorough survey of jackals in both Ethiopia and adjacent countries will be necessary to assess its distribution and abundance of the African wolf. It should be established whether or not the habitat of the African wolf overlap with that of the golden jackal, and if morphological features distinguishing the two taxa exist.

Our results show that *C. a. lupaster* should no longer be accepted as a monophyletic subspecies of *C. aureus* but represents the only grey wolf taxon known to inhabit the African continent.
